# The Prevalence of Hearing Impairment by Age and Gender in a Population-based Study

**Published:** 2017-09

**Authors:** Alimohamad ASGHARI, Mohammad FARHADI, Ahmad DANESHI, Mehdi KHABAZKHOOB, Saman MOHAZZAB-TORABI, Maryam JALESSI, Hesamedin EMAMJOMEH

**Affiliations:** 1. ENT and Head & Neck Research Center, Hazrat Rasoul Akram Hospital, Iran University of Medical Sciences, Tehran, Iran; 2. Skull Base Research Center, Iran University of Medical Sciences, Tehran, Iran; 3. Dept. of Medical Surgical Nursing, School of Nursing and Midwifery, Shahid Beheshti University of Medical Sciences, Tehran, Iran; 4. Noor Research Center for Ophthalmic Epidemiology, Noor Eye Hospital, Tehran, Iran

**Keywords:** Prevalence, Hearing impairment, Epidemiology, Iran

## Abstract

**Background:**

This study aimed to determine the prevalence of hearing impairment (HI) by age and gender in a population aged 5 yr and older residing in Tehran, Iran.

**Methods:**

In this cross-sectional study, 140 clusters each including 10 households from Tehran, Iran were sampled between 2012 and 2013 using cluster random sampling. Trained audiologists examined the participants during face-to-face interviews. The hearing of the participants was evaluated before the removal of wax or other foreign bodies. In this study, HI was categorized as mild (grade 1, 26–40 db), moderate (grade 2, 41–60 db), severe (grade 3, 61–80 db), and deaf (grade 5, 81 db or more). All participants signed informed consent forms. The SATA software was used for data analysis.

**Results:**

Of 6521 individuals, 4370 (67%) were interviewed. The prevalence of HI (auditory threshold of 0.5, 1, 2, 4 KHz and more than 25 db in the better ear) was 14.27 (11.53–17.91) of whom 9.52 (7.07–11.98) had grade 1, 4.04 (3.02–5.06) had grade 2, 0.67 (0.33–1.02) had grade 3 HI and 0.48 (0.16-0.8) were deaf. About 5.19% of the participants had disabling hearing impairment. All HI grades increased significantly with age but no significant difference was observed between men and women.

**Conclusion:**

The considerable prevalence of HI in Iran in comparison with other developing countries, with regards to the trend of aging in the population, seems concerning. The results of the study could be used as a treatment and research guideline for future works in the area of policymaking and plan to decrease these disorders.

## Introduction

The three-fold increase in the elderly population of the world by the year 2050 has made hearing impairment (HI) the third most common disability in the elderly according to the statistics of the WHO (1–3). On the other hand, HI, in addition to comprising 4.7% of the total causes of years lived with disability (YLD) in the world, imposes a great disease burden on the health system due to long-term social, functional, and psychological complications (4, 5). Moreover, the prevalence of HI has been on the rise in all age groups across the world (6). In the USA, the prevalence of HI increased from 14.9% in 1994–1998 to 19.5% in 2005–2006. In another report, its prevalence increased by two times from 1965 to 1994 in the USA (7, 8). Furthermore, HI has affected two-thirds of the 70-year-old Americans and one-third of the Japanese population in the 4th decade of life and half of the Japanese population above 60 yr of age (9, 10). In addition, 14.9% of the American children (more than seven million children) suffer from different types of HI (11). Despite the importance of this issue, few studies were performed on the prevalence of HI and the associated disorders in Iranian children in the late 1990s and as a result, our knowledge of the total prevalence of the different levels of HI in the Iranian population is very limited.

However, a broad spectrum of environmental and genetic factors can contribute to the development of HI in different societies (12). The most important causes of HI are noise-induced hearing loss (13, 14) diabetes (15, 16) and heavy metals (15, 16) in association with genetic and congenital diseases, infectious diseases before and after birth, and drugs (17–19). HI has a great impact on health through overlapping with or causing other diseases like increasing the frequency of depression (20), communication problems, dementia (21) and cognitive disorders (22); therefore, it has a deep impact on the quality of life (23). According to the reports from the US, despite the high prevalence of HI, 36% of the people never undergo hearing evaluation (24). Therefore, WHO intends to prevent the long-term complications of the HI in countries with low and middle-income countries through screening programs (25). Iran is the second most populated country in the Middle East with an aging population; however, few population-based studies have been conducted on hearing disorders in Iran but each one has its own limitation like the study population, sample size, or measurement method (26, 27).

The aim of the present study was to determine the prevalence of HI and deafness in an Iranian population based on the WHO definitions.

## Materials and Methods

This cross-sectional study was performed by the ENT and Head and Neck Research Center of Iran University of Medical Sciences and Iran National Science Foundation between 2012 and 2013 in Tehran, Iran. This study had 2 phases. In the first phase, 140 clusters each including 10 households in individuals above 6 months were sampled using cluster random sampling. Head clusters were also selected randomly according to the 10-digit postal code. In each cluster, sampling was performed for up to 10 households. First, the head cluster household was visited. Then, using a systematic method, 10 nearby households joined the study in a clockwise manner. All examiners were audiometrists that had mastery overhearing tests and were capable of communicating with the participants. In the second phase, after coordination with all households in each cluster, the trained audiometrists attended the participants’ houses and conducted the interviews and audiometric tests. All audiology and ambient noise devices were calibrated with reference devices.

A questionnaire including some demographic and specialized data was completed. This questionnaire was designed by the WHO Ear and Hearing Disorders Survey protocol whose validity and reliability was previously confirmed. All the participants signed informed consent forms.

This study was approved by the Medical Ethics Committee of ENT Research Center.

### Examinations

Preliminary evaluations included the assessment of 1) otalgia, 2) auricle shape (normal or abnormal), 3) the presence of inflammatory factors, wax, foreign body, fungal infection, and otorrhea in the external auditory canal, 4) the presence of tympanic perforation, opacity, protrusion, or inflammation, 5) the presence of otorrhea in the middle ear.

The audiometrists first examined adult participants to reduce the fear of children and younger adults during the examinations. If any foreign body or wax was observed in the external canal, auditory evaluations were performed without their removal in order to assess their effects on hearing. The second set of examinations and audiometry were performed after wax removal at the discretion of the audiometrists.

In individuals above the age of 5 yr, audiometry was performed after the participant received complete explanations. Before audiometry started, a rather quiet room was chosen, the ambient noise was measured, and its level was recorded in the relevant section of the questionnaire. The ambient noise should be preferably less than 40 db according to the WHO protocol. If the ambient noise was more than 40 db, its measurement was done and the result was recorded. On the other hand, the auditory threshold of the participants was measured in the frequencies of 1, 2, 4, and again 1 kHz. At the beginning, hearing in each ear was evaluated in 1 KHz by 60 dB sound level.

If no response was observed, the sound level was increased in 10-dB increments until the desired response was achieved. When the participant responded, the auditory threshold was determined by reducing the intensity by 10 dB and then increasing it by 5 dB with no correcting factor. All these thresholds were examined at the frequencies of 2 and 4 kHz, as well. In the end, the auditory threshold was again examined at 1 kHz; all the steps were repeated if the final threshold measurement at 1 KHz had a difference of more than 5 dB with the primary measurement. The classification of hearing impairment by dB is presented in Table 1. Hearing impairment grade 2 to 4 was categorized as disabling hearing impairment.

**Table 1:** Classification of hearing impairment according to WHO criteria

**Grade of impairment**	**Corresponding audiometric ISO value**	**Performance**	**Recommendations**
**0 - No impairment**	25 dB or better (better ear)	No or very slight hearing problems. Able to hear whispers.	
**1 - Slight impairment**	26–40 dB (better ear)	Able to hear and repeat words spoken in normal voice at 1 meter.	Counselling. Hearing aids may be needed.
**2 - Moderate impairment**	41–60 dB (better ear)	Able to hear and repeat words spoken in raised voice at 1 meter	Hearing aids usually recommended.
**3 - Severe impairment**	61–80 dB (better ear)	Able to hear some words when shouted into better ear.	Hearing aids needed. If no hearing aids available, lip-reading and signing should be taught.
**4 - Profound impairment including deafness**	81 dB or greater (better ear)	Unable to hear and understand even a shouted voice.	Hearing aids may help understanding words. Additional rehabilitation needed. Lip-reading and sometimes signing essentially.

After data collection was completed, the participants were divided into the following age groups: 5–10 yr, 11–20 yr, 21–30 yr, 31–40 yr, 41–50 yr, 51–60 yr, 61–70 yr, and more than 70 yr.

### Data Analysis

The SATA software was used for data analysis. We reported the prevalence of HI as percentage with a 95% confidence interval (CI). To calculate CI, the effect of cluster sampling was regarded. Logistic regression was used to evaluate the correlation of HI with age and gender and the odds ratios were reported.

## Results

Of the 6521 selected individuals, 4370 (67.0%) participated in the study of whom 4213 were 5 yr and older and 2280 (54.1%) were female.

The total prevalence of HI was 14.72% (95% CI 11.53–17.91). Moreover, 64.71%, 27.45%, 4.58%, and 3.27% of the hearing impairment participants had HI grade 1, 2, and 3 and 4 (deafness), respectively. Table 2 presents the prevalence of different HI grades by gender. The prevalence of deafness was 0.48% (95% CI 0.16–0.8) in the present study and 5.19% of the participants had to disable HI. Evaluation of the correlation between the prevalence of HI between male and female participants using logistic regression showed that the total prevalence of HI and the prevalence of HI grade 1 and 2 were significantly higher in males. The results of logistic regression are presented in Table 1.

**Table 2:** The prevalence of hearing impairment by grade according to gender

	**Total**	**Female**	**Male**	**OR(95%CI), *P*-value**
**Hearing impairment**	14.72(11.53−7.91)	12.6(9.55−5.66)	17.74(13.83−1.65)	1.5(1.24–1.8), <0.001
**Grade 1=slight**	9.52(7.07–11.98)	8.1(5.57–10.63)	11.55(8.63–14.48)	1.48(1.14–1.93), 0.004
**Grade 2=moderate**	4.04(3.02–5.06)	3.36(2.32–4.39)	5.02(3.41–6.63)	1.52(1.03–2.24), 0.035
**Grade 3=severe**	0.67(0.33–1.02)	0.65(0.19–1.12)	0.7(0.16–1.24)	1.07(0.36–3.14), 0.901
**Grade 4=deaf**	0.48(0.16–0.8)	0.49(0.09–0.89)	0.47(0.03–0.91)	0.95(0.3–3.04), 0.931
**Disabling hearing impairment (grad 2, 3 and 4)**	5.19(4.05–6.34)	4.5(3.29–5.71)	6.18(4.4–7.96)	1.4(0.98–2), 0.065

**Fig. 1:**
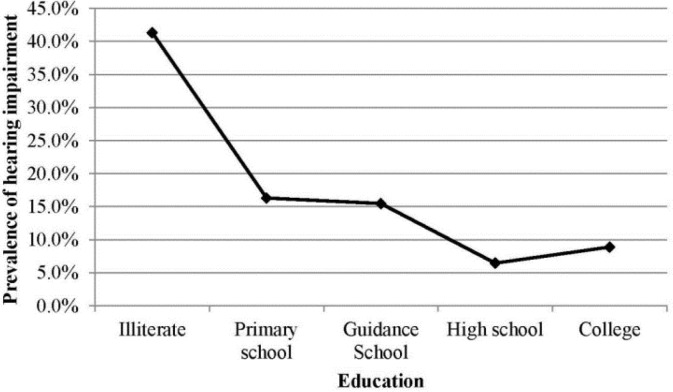
The prevalence of hearing impairment according to the educational level

Table 3 shows the prevalence of HI in different age groups. According to Table 2, the prevalence of HI increased in all levels by aging. Table 4 presents the association of HI with age and gender in a multiple models. In this model, the prevalence of hearing impairment had no significant association with gender while all grades of HI increased significantly with age. Fig. 1 shows the prevalence of HI according to the educational level. The prevalence of HI decreased significantly from 41.3% in illiterate participants to 8.9% in participants with university education (*P*<0.001).

**Table 3:** The prevalence (%) of hearing impairment in different age groups

	**Hearing impairment**	**Grade 1=slight**	**Grade 2=moderate**	**Grade 3=severe**	**Grade 4=deaf**	**Disabling hearing impairment (grad 2, 3 and 4)**
**5–10**	0.6	0.0	0.6	0.0	0.0	0.6
**11–20**	1.9	1.7	0.0	0.0	0.3	0.3
**21–30**	3.3	2.4	0.6	0.3	0.0	0.9
**31–40**	3.6	2.9	0.7	0.0	0.0	0.7
**41–50**	7.4	6.8	0.3	0.0	0.3	0.6
**51–60**	20.1	14.0	5.0	0.7	0.4	6.1
**61–70**	42.6	31.2	9.9	1.0	0.5	11.4
**>= 71**	70.4	33.3	27.7	5.7	3.8	37.1

**Table 4:** The association of hearing impairment with age and gender in a multiple logistic regression model

		**OR(95% CI)**	***P*-value**
**Hearing impairment**	Sex	1.16(0.89–1.5)	0.264
	Age	1.09(1.07–1.12)	<0.001
**Grade 1=slight**	Sex	1.21(0.89–1.64)	0.218
	Age	1.06(1.04–1.09)	<0.001
**Grade 2=moderate**	Sex	1(0.62–1.62)	0.988
	Age	1.1(1.08–1.12)	<0.001
**Grade 3=severe**	Sex	0.67(0.2–2.3)	0.525
	Age	1.1(1.04–1.15)	<0.001
**Grade 4=deaf**	Sex	0.67(0.21–2.1)	0.489
	Age	1.07(1.02–1.13)	0.01
**Disabling hearing impairment (grad 2, 3 and 4)**	Sex	0.9(0.58–1.41)	0.651
	Age	1.1(1.08–1.12)	<0.001

## Discussion

The WHO has provided executive protocols for the collection of the data of hearing disorders in regional and provincial level, for epidemiologic studies on hearing and other ear disorders. In this study, for the first time in Iran and as the second country in the Middle East, we used the WHO protocol to evaluate the prevalence and causes of HI in an Iranian population.

According to our results, 14.7% of the society experienced some levels of HI and about two-thirds of them (9.9%) had mild HI (grade 1). Table 5 presents the prevalence of HI in different countries. For the first time in the Middle East, the prevalence of hearing impairment was reported 5.5% and 36.06 in 1000 population in Oman in 2004. Despite the two-fold prevalence of hearing impairment in our country, only one-third of the participants had mild HI in Oman (28).

**Table 5:** The prevalence of hearing impairment and deafness in different countries

**Country**	**Year**	**n**	**Age**	**Prevalence of Deafness (or profound hearing loss)**	**Prevalence of Hearing Impairment**
				**Total**	
**USA (35)**	2015	16415	18–74	-	15.06%
**Brazil (31)**	2007	2427	4<	-	26.1%
**Bangladesh (44)**	2014	3707	>18		0.3/1000
**China (45)**	1993	-	-	0.186	-
**USA (46)**	1998	-	3–10	1.1/1000	-
**France (47)**	1996	-	<9	.54/1000	-
**Australia (48)**	2011	3258	21–84	-	14.1%
**USA (49)**	1998	3753	48–92	45.9%	-
**USA (7)**	2010	2005–2006=1771	12–19	19.5%	-
		1988–1994=2928		14.9%	
**Italy (50)**	1998	2398	>65	-	19%
**Australia (51)**	2007	2431	Mean= 67.0	-	44.6%
**USA (11)**	1998	6166	6–19	14.9%	-
**USA (52)**	2004	White= 107100	>18	-	11.0%–12.7%
		African-American= 17904		-	5.9%–8.5%
**USA (53)**	2005	2052	73–84	59.9%	-
**Oman (54)**	2010	1639	>60	3.6%	33.5%
**Oman (55)**	2004	12400	-	-	-
**USA (56)**	2006	>5 y/o population	>5	4.1%(41 per 1000 or 11,000,00)	-
**Egypt (29)**	2007	4000	-	16.0%	-
**Uganda (57)**	2008	6041	-	-	Child=10.2% Adult=11.7%
**Global (57)**	2013	-	5–14 y/o	-	1.4%
			Female >15 y/o		9.8%
			Male >15 y/o		12.2%
**China (58)**	2006	1261	>60	1.3%	58.1%
**Korea (59)**	2014	18650	-	-	22.73%

In another research on 4000 participants in different parts of Egypt in the Middle East, the prevalence of hearing loss was reported about 16%; considering the social texture and population of Iran and Egypt, a rather similar prevalence of HI was expected (29). The prevalence of HI is higher in Taiwan and Brazil than Iran (21.4 and 26.1%, respectively) (30, 31). The reports from developed countries are very different; for example, the prevalence of hearing impairment has been reported 16.9%, 16%, 26.7%, 4%, and 15.06% in Sweden (6), England (32), Norway (33), Canada (34), and the US (35), respectively.

On average, the prevalence of HI in developed countries (4.9%) is much lower than its prevalence in Africa (15.7%) and South Asia (17.0%) (25). A broad spectrum of diseases including genetic factors (6), pre and postnatal infections (36), otitis media (28), and foreign body (37) cause hearing disorders in children and adults. However, most studies have eliminated the children age group and defined their target population as adults. The sampling method is also important when evaluating HI in different societies; in our study, hearing evaluation was performed prior to the removal of wax or foreign body while this process has been performed after wax removal in many studies. Moreover, the type of the target population (urban, rural, developed, and developing) and other factors such as level of health care and lifestyle should also be considered when evaluating hearing problems.

In the present study, the prevalence of the level of HI increased in both sexes with age; 1% of the participants aged 5–10 yr had HI while more than two-thirds of the people above the age of 70 experienced auditory disorders. Other studies have also reported similar findings (38, 39); for example in China, the prevalence of HI is 3.28% in the society and 12.8% at the age of 60 yr (6). Sixty percent of the people with hearing loss had a mean age of 75.5 yr (40).

The trend of exacerbation of hearing disorders with age can be explained by personal and environmental factors. Presbycusis, exposure to Environmental factors as ototoxic materials and drugs (41) and noise pollution (42) increase the incidence of hearing loss at older ages. On the other hand, according to our findings and the findings of other studies (43) the lower prevalence of HI in the educated people and in the young population versus the elderly population, due to higher levels of education in the young, is expected. However, HI might have resulted in the lack of academic progress. Nevertheless, the prevalence of hearing disorders was higher in our elderly participants when compared to other studies, which may be due to environmental factors although the age cohort effect may also play a role in this regard.

In spite of the fact that no association was found between gender and HI in our study and a study performed in Italy (60), previous studies have published different reports indicating an increase in the prevalence of these disorders in men (33, 61–63) and women (64–66).

This study had some weak and strong points mentioned. The most important strong point of the study was determining the prevalence of visual disorders in a population-based study with a large sample size using cluster sampling. The limitations of this study was an attrition of about 33% during sampling and a response rate of 67%, resulted in selection bias. Therefore, attention should be paid to the bias.

## Conclusion

The considerable prevalence of HI in Iran in comparison with other developing countries, with regards to the trend of aging in the population, seems concerning. This study is the first epidemiologic study of hearing loss in the national level and its results could be used as a baseline for other researches and evaluation of burden of HI in our country.

## Ethical considerations

Ethical issues (Including plagiarism, informed consent, misconduct, data fabrication and/or falsification, double publication and/or submission, redundancy, etc.) have been completely observed by the authors.
